# The complete coding sequence of a rabies lyssavirus (RABV) detected in an American black bear (*Ursus americanus*) in Connecticut, USA.

**DOI:** 10.1128/MRA.00821-23

**Published:** 2024-02-13

**Authors:** Zeinab H. Helal, Nina Francesca Soriano, Ji-Yeon Hyeon, Hyunjung Chun, Maureen Sims, Amelia Wheeler, Guillermo R. Risatti

**Affiliations:** 1 Connecticut Veterinary Medical Diagnostic Laboratory, Department of Pathobiology and Veterinary Science, College of Agriculture, Health and Natural Resources, University of Connecticut, Storrs, Connecticut, USA; 2 College of Veterinary Medicine, Konkuk University, Seoul, South Korea; Katholieke Universiteit Leuven, Leuven, Belgium

**Keywords:** rabies virus, genome, black bear, Connecticut, next-generation sequencing, molecular epidemiology, wild rabies

## Abstract

The complete coding sequence of a rabies lyssavirus (RABV) detected in a black bear (*Ursus americanus*) was generated. RNA extracted from brain tissues was amplified using reverse transcription followed by tiling PCR sequencing to obtain RABV whole viral genome. Sequencing was performed using an Illumina ISeq 100 instrument.

## ANNOUNCEMENT

Rabies virus (RABV) belongs to the order *Mononegavirales*, family *Rhabdoviridae*, genus *Lyssavirus*, and species *Lyssavirus rabies*. The complete coding sequences of an RABV detected in black bears (*Ursus americanus*) are not available.

A wild adult female black bear (Sample ID 23–306) was submitted for necropsy to the Connecticut Veterinary Medical Diagnostic Laboratory (CVMDL) at the University of Connecticut in March 2023. The bear was reported to be lying down not responding to human presence. The bear did not appear to be in a den and had mobility difficulties affecting the left side of the body. The animal was observed for an additional 24 hours and then euthanized by a Connecticut conservation officer. The brain of the bear was tested for RABV using direct fluorescent antibody (DFA) testing (1). The DFA test was performed by following the CDC’s “Protocol for Postmortem Diagnosis of Rabies in Animals by Direct Fluorescent Antibody Testing.” RABV was detected in the brain stem, cerebellum, and hippocampus. Total RNA was then extracted from the brain stem using the Trizol reagent (ThermoFisher Scientific, Waltham, MA, USA). A total of 100 mg of brain tissue was placed in a tube containing 0.1 mm zirconia grinding media (Glen Mills, Clifton, NJ, USA) and 1 mL TRIzol reagent and homogenized in a Biospec Mini Beadbeater (Biospec, Bartlesvilly, OK, USA) for 1 minute at 4,800 rpm.

The enhanced multiplex tiling RT-PCR protocol was used to amplify the complete coding sequence of the virus as was done in a previous study (2).

A multiplexed paired-end sequencing library of the PCR products was generated using the Nextera DNA Flex Library Prep Kit (Illumina, Foster City, CA, USA). The library was adjusted to 100 pM, and 5% of the PhiX control (Illumina) was added to the library. The library pool was loaded into an ISeq100 i1 Reagent Kit (Illumina). The barcoded multiplexed library sequencing (2 × 151 bp) was performed on an Illumina iSeq100 platform. Fastq files were uploaded to Galaxy (3) and utilized in batch mode to recognize and run pairs together. Residual adapters and bases with low-quality scores were removed using Trimmomatic version 0.38.0 (4) removing bases from each read with a quality score <Q 20 and required a minimum read length of 50 bases each. The total number of reads is 3,582,574 and the sequencing depth is 97.7. The GC content is 45.4%.

**Fig 1:**
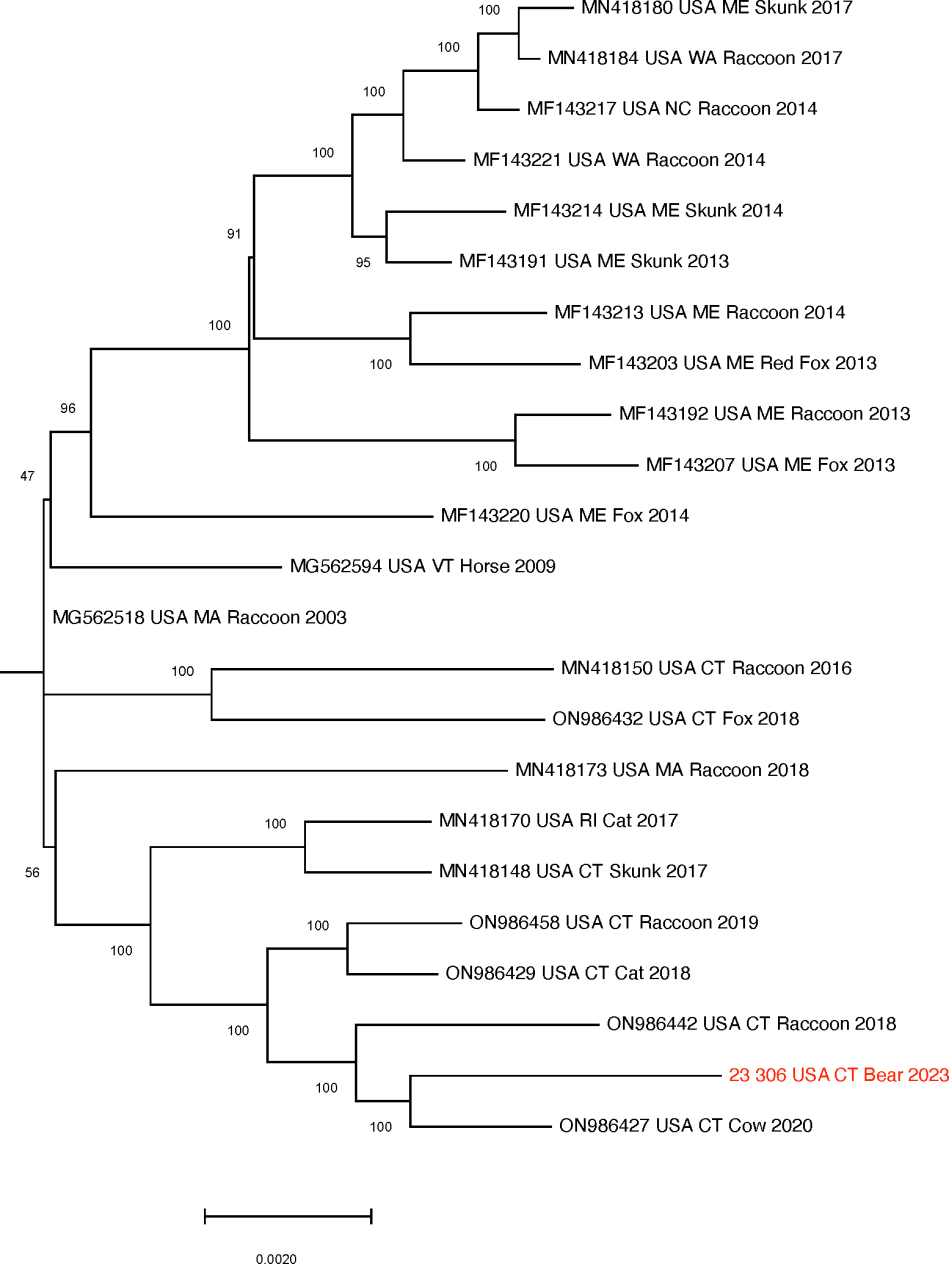
Expansion of the clade of 23_306_USA/CT_bear from the full maximum-likelihood tree. The scale bars show the number of substitutions per site. The software MAFFT multiple alignment Version: 1.4.0 in Geneious was used for sequence alignment of whole-genome sequences. Maximum-likelihood (ML) phylogeny was constructed using RAxML-HPC v.8 with 1,000 bootstrap replicates on XSEDE with default parameter settings. The phylogeny was rooted at the midpoint. The scale bars show the number of substitutions per site. The numerical values represent 1,000 bootstrap replicate values expressed as a percentage.

Trimmed reads were assembled using the SPAdes assembler from Galaxy Shovill version 1.0 (5) and the consensus genome sequences were called using the Geneious Prime 10 with default parameter settings. The contig was 12,097 bp in length, hereafter referred to as 23_306_USA/CT_bear. For phylogenetic analysis, a total of 22 whole-genome sequences of RABVs were downloaded from the NCBI GenBank database (Fig. 1). RABV 23_306 USA/CT_bear clustered with cow and raccoon viruses. The two closest RABVs detected in a cow and a bear (Fig. 1) have 99.3% nucleotide similarity.

Rabid black bears have been detected in Canada (6) although the sequences of this virus have not been reported.

## Data Availability

This Whole-Genome Shotgun work has been deposited in the GenBank under accession no. OR474090.1 Lyssavirus rabies isolate 23_306_USA/CT_bear, complete genome (https://www.ncbi.nlm.nih.gov/nuccore/2588207170). The version described in this paper is the first version, OR474090.1. Sequence data have been deposited in the Sequence Read Archive (SRA), under BioProject accession number PRJNA1052630, with a release date: 2023-12-14.
